# New Perspectives in Treating Acute Myeloid Leukemia: Driving towards a Patient-Tailored Strategy

**DOI:** 10.3390/ijms23073887

**Published:** 2022-03-31

**Authors:** Fabio Andreozzi, Fulvio Massaro, Sebastian Wittnebel, Chloé Spilleboudt, Philippe Lewalle, Adriano Salaroli

**Affiliations:** 1 Hematology Department, Institut Jules Bordet, Université Libre de Bruxelles, 1000 Bruxelles, Belgium; fulvio.massaro@bordet.be (F.M.); sebastian.wittnebel@bordet.be (S.W.); chloe.spilleboudt@bordet.be (C.S.); philippe.lewalle@bordet.be (P.L.); adriano.salaroli@bordet.be (A.S.); 2 PhD Program in Clinical and Experimental Medicine, University of Modena and Reggio Emilia, 41121 Modena, Italy

**Keywords:** acute myeloid leukemia, immunotherapy, target therapy, venetoclax

## Abstract

For decades, intensive chemotherapy (IC) has been considered the best therapeutic option for treating acute myeloid leukemia (AML), with no curative option available for patients who are not eligible for IC or who have had failed IC. Over the last few years, several new drugs have enriched the therapeutic arsenal of AML treatment for both fit and unfit patients, raising new opportunities but also new challenges. These include the already approved venetoclax, the IDH1/2 inhibitors enasidenib and ivosidenib, gemtuzumab ozogamicin, the liposomal daunorubicin/cytarabine formulation CPX-351, and oral azacitidine. Venetoclax, an anti BCL2-inhibitor, in combination with hypomethylating agents (HMAs), has markedly improved the management of unfit and elderly patients from the perspective of improved quality of life and better survival. Venetoclax is currently under investigation in combination with other old and new drugs in early phase trials. Recently developed drugs with different mechanisms of action and new technologies that have already been investigated in other settings (BiTE and CAR-T cells) are currently being explored in AML, and ongoing trials should determine promising agents, more synergic combinations, and better treatment strategies. Access to new drugs and inclusion in clinical trials should be strongly encouraged to provide scientific evidence and to define the future standard of treatment in AML.

## 1. Introduction

Acute myeloid leukemias (AMLs) comprise a heterogeneous group of conditions that are characterized by the uncontrolled proliferation of a transformed malignant hematopoietic myeloid cell, inevitably leading to bone marrow failure.

Intensive chemotherapy (IC), consisting of anthracycline and cytarabine in induction treatment, high or intermediate doses of cytarabine as consolidation followed by allogeneic stem cell transplantation (HSCT) in high/intermediate-risk patients, has been considered as the gold standard in the treatment of AML for decades.

However, with a median age at diagnosis of 68 years, a high percentage of AML patients are not eligible for such an intensive therapeutic program [1]. Moreover, AML with myelodysplastic-related changes and therapy-related AML are associated with poor prognostic cytogenetic features, such as a complex karyotype and TP53 mutation, and are more frequent in the elderly; they show very unsatisfactory response rates to conventional IC, making it suitable neither for all patients nor for all diseases [2].

In recent decades, the hypomethylating agents (HMAs) azacitidine and decitabine have become the preferred low-intensity options in AML treatment for unfit patients, allowing about one-third of treated patients to reach remission and transfusion independence, improving their overall survival (OS) and quality of life [3,4,5]. In particular, azacitidine has shown better outcomes compared to IC in poor cytogenetic risk patients and less myelotoxicity compared to decitabine [6,7].

Recent advances in the genetic characterization of AML and in understanding the molecular mechanisms at the basis of leukemogenesis has allowed for a better prognostic assessment of the disease and opened the way to the exploration of new tailored therapeutic strategies based on patients’ risk profiles. 

In parallel, owing to advances in drug manufacturing, bioengineering, and cellular therapy, more potent and selective agents and more advantageous formulations of old drugs are now available.

This progress is driving AML treatment from a ‘one fits all’ strategy to a precision therapy dimension, providing a real paradigm shift in the treatment of leukemia patients.

With this work, we aimed to write a narrative review of the recent advances in AML treatment and describe the landscape of future possibilities in this setting. 

The most relevant papers were chosen after an extensive PubMed query; oral communications were selected from international hematology congresses (ASH and EHA congress). The main keywords used in the search strategy were adult acute myeloid leukemia, new drugs, target therapy, and immunotherapy.

## 2. Harnessing the Apoptosis Pathway

**Figure 1:**
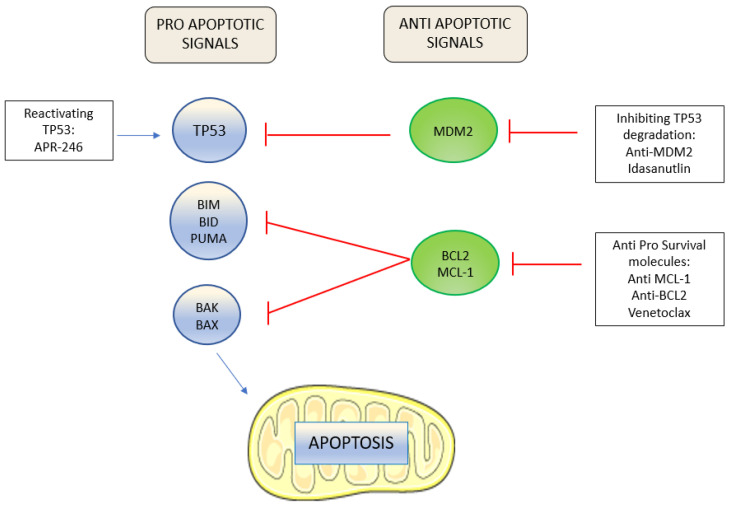
Pro and anti-apoptotic signals and drugs acting on the intrinsic apoptosis pathway. Proapoptotic signals are counterbalanced by anti-apoptotic signals to promote cell survival. Drugs can induce apoptosis through the reactivation of mutated TP53, the inhibition of TP53 degradation, and by blocking pro-survival molecules.

The B-cell lymphoma 2 (BCL-2) family proteins finely regulate the intrinsic cellular apoptosis pathway (Figure 1). In normal conditions, cell death is prevented by the BCL-2 family anti-apoptotic proteins BCL2 and MCL1 through the sequestration of pro-apoptotic molecules.

In response to cell stress or damage, the pro-apoptotic members of the BCL-2 family, BID and BIM, sensitized by BH3-only proteins, interact with the effector proteins BAX and BAK to provoke mitochondrial membrane permeabilization and caspase release, and, consequently, cell apoptosis [8].

TP53 plays a key role in the intrinsic apoptosis pathway: it is activated in stress conditions, for example, with DNA damage induced by chemotherapy, and triggers apoptosis mainly by promoting the transcription of BH3-only proteins [9].

A better understanding of mechanisms at the basis of the cellular apoptosis pathway prompted the development of different classes of molecules with anti-leukemic activity.

### 2.1. BCL Inhibitors—Venetoclax

First identified in follicular lymphoma (FL), the anti-apoptotic protein BCL-2 prevents cell death by binding the pro-apoptotic BAX/BAK proteins and protecting the integrity of the outer mitochondrial membrane [10,11,12].

BCL-2 has been found to be overexpressed in AML cells and to confer resistance to conventional chemotherapy [13,14].

While the anti-sense oligonucleotide oblimersen and the first BH3 mimetic obatoclax showed limited efficacy in early clinical trials, the oral BCL-xL and BCL-2 inhibitor navitoclax showed significant anti-tumoral activity in preclinical AML cells and xenograft models [15,16]. Navitoclax was subsequently tested in patients affected by solid tumors and chronic lymphocytic leukemia (CLL), confirming its preclinical efficacy but also significant hematological toxicity, mainly thrombocytopenia [17]. To overcome this effect caused by the inhibition of BCL-xL by navitoclax, the BCL-2 selective inhibitor venetoclax was developed [18,19].

Venetoclax is an oral, highly selective, and potent BCL-2 inhibitor, first employed in CLL treatment [19,20]. By binding the BCL-2 protein, venetoclax allows for the release of pro-apoptotic proteins, restoring the apoptosis pathway [21,22]. 

After preclinical studies confirmed the pro-apoptotic activity of venetoclax in AML cell lines, it was administered as monotherapy in relapsed or refractory (R/R) AML patients, where it showed a tolerable profile but modest clinical efficacy, suggesting the need to explore its potential combinations with other agents, particularly HMAs [23,24].

Venetoclax is already used in clinical combination with HMAs in AML patients who are not eligible for IC, and many associations with other agents (IC and new drugs) are currently under exploration (Table 1). 

#### 2.1.1. Venetoclax in Association with Hypomethylating Agents

Preclinical data has shown a synergistic effect of the combination of venetoclax with HMAs and the association of venetoclax with azacitidine/decitabine has been proven to be safe and able to produce a favorable response even in high-risk AML patients in early trials [25,26].

In the phase III study VIALE-A, azacitidine and venetoclax produced significantly better OS (14.7 versus 9.6 months), complete remission (CR) rates (37% vs. 18%), and composite remission rate (CR + CRi; 66% versus 28%) compared to azacytidine alone in AML patients who had not been previously treated and who were ineligible for IC [27].

Based on the VIALE-A study results, venetoclax received full approval from the Food and Drug Administration (FDA) in October 2020 in combination with azacitidine, decitabine, or low-dose cytarabine (LDAC) for the treatment of newly diagnosed (ND) AML in patients 75 years or older or in patients with comorbidities precluding the use of IC. Similarly, the European Medical Agency (EMA) approved the use of venetoclax in combination with an HMA for IC-ineligible adult AML patients.

In the R/R setting, no randomized controlled trials are available; however, retrospective data show interesting results with ORR about three times higher than the conventional salvage treatment with a single HMA, allowing in some cases to proceed to allogeneic stem cell transplantation (ASCT) [28,29].

In one study, a ten-day decitabine schedule was associated with venetoclax and compared to standard IC in 65 patients with R/R AML; the experimental arm showed a better ORR (60% vs. 36%, respectively), minimal residual disease (MRD) negativity by flow cytometry (28% vs. 13%), longer event-free survival (EFS; 5.7 vs. 1.5 months), and OS (6.8 vs. 4.7 months) [30].

The combination of azacitidine or decitabine with venetoclax can also be effective in AML relapse following ASCT, as shown in a retrospective analysis of 32 R/R patients conducted by the German Cooperative Transplant Study Group that reported an ORR of 47%, of which 50% were CR [31]. 

#### 2.1.2. Venetoclax in Combination with Low-Dose Ara-C

The phase III study VIALE-C compared venetoclax plus low dose Ara-C (LDAC) with placebo plus LDAC in ND AML patients who were ineligible for IC [32].

Although no statistically significant differences were observed in OS (7.2 for the combination of LDAC and venetoclax versus 4.1 months in the control arm), and thus the primary endpoint of the study was not met, venetoclax provided a 25% reduction in the risk of death over the LDAC alone arm. A higher rate of CR/CRi was observed with venetoclax plus LDAC vs. LDAC alone: 48% vs. 13% with CR achieved in 27% vs. 7% of the patients, respectively. A post-hoc analysis with an additional 6 months of follow-up reported a median OS for the venetoclax and placebo arm of 8.4 and 4.1 months, respectively [33].

#### 2.1.3. Venetoclax in Association with Intensive Chemotherapy

Considering the improved outcome when added to the treatment of elderly or unfit AML patients, venetoclax is under evaluation for younger and fit patients in addition to different IC regimens.

Thus far, no published data is available concerning the association of the standard IC backbone (7 + 3) to venetoclax. A phase Ib trial (NCT03709758) enrolling ND AML is ongoing and a phase III randomized placebo-controlled 7 + 3 and venetoclax in ND AML or MDS-EB will be recruiting soon (NCT04628026).

In a single arm, phase II trial at the MD Anderson Cancer Center, CLIA (cladribine plus high-dose aracytin and idarubicin) was associated with venetoclax in ND AML, higher risk MDS, and mixed phenotype acute leukemia patients. Composite complete response (CCR) was achieved by 94% of patients, with 82% having undetectable MRD. At a median follow-up of 13.5 months, the median duration of response (DOR), EFS, and OS were not reached. The toxicity profile was excellent, with only two deaths in CR occurring in patients receiving a concomitant FLT3 inhibitor (FLT3i) [34].

In a phase Ib dose escalation study, venetoclax was associated with a 5 + 2 regimen in 51 elderly AML patients who were ineligible for IC; the authors reported noteworthy CR/CRi rates (72% in the whole population with 94% in de novo and 43% in secondary AML) and a median OS of 11 months. An impressive marrow blast reduction ≥ 50% was observed in NPM1-, IDH2-, and SRSF2-mutant AML during the venetoclax monotherapy pre-phase [35].

Di Nardo and colleagues published very promising results of a phase Ib/II trial of venetoclax in association with FLAG-Ida in ND and R/R AML [36]. These results were recently updated at ASH 2021: the CRc rate was 88%, with 92% of this group achieving an MRD-negative status, and 66% of patients bridged to ASCT. The toxicity profile was acceptable, with febrile neutropenia (39%) and pneumonia (24%) being the most frequent adverse events (AEs). With a median follow-up of 16 months, median OS and EFS were both not reached, with 1-year OS and EFS rates of 96% and 77%, respectively. The presence of a TP53 mutation at diagnosis conferred a significantly inferior outcome compared to wild type (WT) status, with an OS of 24 months compared to not reached (*p* = 0.03) and an EFS of 8 months compared to not reached in the two groups (*p* = < 0.001), respectively [37].

All patients with a TP53 mutated at diagnosis inevitably relapsed, and two patients who were initially TP53 WT relapsed as TP53 mutated [38].

Novel associations are still under evaluation, such as CPX 351 plus venetoclax in ND AML without FLT3 or IDH mutations (NCT04075747) or a low-intensity dose of CPX 351 plus venetoclax in ND AML patients who are ineligible for IC. These studies are currently recruiting and thus have no available data concerning efficacy and safety.

#### 2.1.4. Venetoclax—Future Non-Intensive Combinations

Considering the efficacy and tolerability of combinations based on venetoclax and the existence of driver mutations for which several drugs were recently approved, venetoclax is being actively studied in combination trials. 

The use of multiple drug combinations could likely offer deeper and prolonged responses, owing to synergism and the simultaneous activity on different subclones of AML. The non-overlapping toxicities of different agents could also account for safe and well-tolerated combinations in the elderly and in frail patients [39].

#### 2.1.5. Venetoclax in Association with FLT3 Inhibitors 

FLT3 and ITD mutations in AML seem to confer resistance to venetoclax treatment by enhancing the expression of anti-apoptotic proteins (BCL-xL and MCL1), explaining the relatively weak response to venetoclax-based regimens noted in patients harboring these alterations [26,40,41].

The development of FLT3is prompted an assessment of their efficacy in combination with venetoclax and as part of triple combinations in this unfavorable subgroup of AML patients.

In a phase I study, the association of venetoclax and gilteritinib showed a desirable CR rate (85.4%) in 41 R/R AML patients with an FLT3 mutation, persisting also in patients already exposed to FLT3is [42].

In a phase I/II trial, the combination of azacitidine, venetoclax, and gilteritinib was evaluated in R/R AML, ND AML unfit for IC, or high risk CMML/MDS FLT3-ITD or TKD-mutated AML. This triplet resulted in a CRc rate of 100% and 69% for de novo and R/R patients, respectively [43].

A phase II trial explored the use of a combination of decitabine, venetoclax, and FLT3is (gilterinib, sorafenib, and midostaurin) in 25 ND or R/R FLT3-mutated AML patients. It should be noted that eight R/R patients were previously exposed to FLT3is and four patients previously underwent ASCT [39].

In ND and R/R patients, the CCRs were 92% and 62%, respectively, with more than 50% of responders achieving MRD negativity in both groups. The median OS was 14.5 and 6.8 months in ND and R/R patients, respectively.

The median DOR was not reached in either ND or R/R patients. Nine patients were able to proceed to ASCT: four patients in the ND cohort and five in the R/R cohort.

A triplet combination of decitabine, venetoclax, and quizartinib was evaluated in 17 FLT3-mutated AML patients (13 R/R and four ND) who were ineligible for IC. Significant responses were achieved in both the ND (CR 100%, with 4/4 FLT3-PCR MRD negativity and 2/3 flow cytometry MRD negativity) and R/R group (CR 69% with 4/9 FLT3-PCR MRD negativity and 5/9 flow cytometry MRD negativity). The sixty-day mortality rate was 8% in the R/R setting and 0% in the ND group [44].

#### 2.1.6. Venetoclax in Association with IDH Inhibitors

In accordance with preclinical observations reporting an increased susceptibility to venetoclax in IDH1/2-mutated AML cells, the subgroup of AML patients harboring IDH1/2 mutations has shown high rates of durable remissions in clinical studies associating HMAs with venetoclax. Recently, Pollyea et al. reported a CCR of 79%, a median duration of remission of 29.5 months, and an OS of 24.5 months in ND IDH1/2-mutated AML [27,45,46,47].

A triplet strategy combining ivosidenib and venetoclax with or without azacitidine in 25 patients with IDH1-mutated myeloid malignancies was evaluated in a phase Ib/II study; it showed an acceptable toxicity profile and high rates of MRD-negativity in AML patients [48]. 

The combination of venetoclax and enasidenib is under investigation in patients with IDH2-mutated myeloid malignancies in a phase Ib/II study (NCT04092179); preliminary results from 11 patients, mainly R/R AML, reported a CR + CRi rate of 55% with a tolerable toxicity profile [49].

#### 2.1.7. Other Associations

Other associations with venetoclax are being explored in phase I–II trials, for example, with anti-CD47 antibodies, such as magrolimab, BET-inhibitors, and antibody–drug-conjugated anti CD-123 (NCT03113643, NCT04086264) [50,51].

### 2.2. Anti-Myeloid Leukemia Cell Differentiation Protein-1 (MCL-1)

MCL-1 (myeloid leukemia cell differentiation protein-1) is a pro-survival protein that is implicated in the intrinsic apoptosis pathway, acting by neutralizing BH3-only proteins and preventing mitochondrial membrane permeabilization and consequent cell death [52].

High levels of MCL-1 expression are induced by FLT3 mutations in AML and are correlated with venetoclax resistance, leukemia relapse, and poor outcomes, suggesting a prominent role of MCL-1 as an anti-apoptotic protein in AML [53,54,55].

Preclinical studies have shown that anti-MCL1 treatments have a synergistic activity with venetoclax in AML cells. Moreover, venetoclax-resistant cells keep their susceptibility to MCL-1 inhibition, allowing for the restoration of venetoclax sensitivity [56,57].

MCL1 inhibitors, such as AMG-176 (NCT03797261), AZD5991 (NCT03218683), and S64315 in combination with venetoclax (NCT03672695), are currently under exploration in patients with R/R hematological malignancies.

## 3. Reactivating TP53

TP53 mutations occur in about 10% of AML patients and confer a poor prognosis and refractoriness to conventional IC. The response to HMAs in TP53-mutated patients is less impaired; hence, this class of agents is preferable as a first-line treatment option in this setting. However, the durable response is dismal and long-term survival after ASCT is uncommon, making TP53-mutated AML a disease with an unmet therapeutic need [30,58,59,60]. 

### 3.1. Eprenetapopt (APR-246)

Eprenetapopt (APR-246) induces a conformational change in mutated TP53 that confers thermostability and restores the protein’s native onco-suppressive function [61].

After demonstrating its safety and good tolerability in monotherapy, a phase Ib/II study of a combination with azacitidine provided encouraging remission rates (CR of 44%) in refractory AML patients [62,63,64].

A phase III trial comparing azacitidine plus eprenetapopt to azacitidine alone in patients with TP53-mutated MDS is currently ongoing. (NCT03745716).

### 3.2. Murine Double Minute 2 (MDM2) Inhibitors

Murine double minute 2 (MDM2) is an E3 ubiquitin ligase that degrades TP53. MDMX is an inhibitor of TP53 transactivation [65,66].

In WT TP53 AML, the overexpression of MDM2 and MDMX causes the inactivation of TP53, providing a very strong rationale for the use of MDM2/MDMX inhibitors in synergistic strategies and as venetoclax resensitizing agents [67].

The development of inhibitors acting on both MDM2 and MDMX has encountered some difficulties related to the inadequate size of the molecules and their limited bioavailability. Nevertheless, some MDM2 inhibitors have been developed [68].

RG7112 was the first selective MDM2 inhibitor that showed the capacity to restore TP53 activity and clinical efficacy in R/R AML patients in phase I studies, as monotherapy and in combination with LDAC [69,70,71]. 

Idasanutlin (RG7388) is a second-generation MDM2 inhibitor that is more potent, more selective, and has a more predictable pharmacokinetic profile than RG7112 [72,73].

After some encouraging results from idasanutlin in a phase I study, the phase III study MIRROR failed to meet the primary endpoint of survival benefit for the combination of idasanutlin and cytarabine compared to cytarabine alone [74,75].

Idasanutlin was studied in combination with venetoclax in a phase I study in the upfront treatment of R/R AML patients unfit for IC. This combination was associated with an ORR of 41%, a median time to response of 1.4 months, and a median DOR of 4.9 months [76].

Other small MDM2 inhibitors are currently undergoing clinical investigation, including MK8242 in monotherapy, AMG323 in combination with trametinib and decitabine, DS3032s in monotherapy and in association with azacitidine (NCT02319369), quizartinib, and cytarabine (NCT03552029) [77,78,79].

ALRN-6924, an MDM2-MDMX inhibitor, is under investigation in monotherapy and in combination with cytarabine in a phase I study [80].

Preclinical and clinical research on targeting MDM2 and MDMX is a current field of great interest in the treatment of AML. Challenges for MDM2 inhibitors and anti-MDM2-MDMX development include defining the optimal dose, finding the optimal combination, managing hematological and gastrointestinal toxicities, and defining which type of patients could potentially reap the greatest benefits from them [81].

## 4. Harnessing Immunity

**Figure 2:**
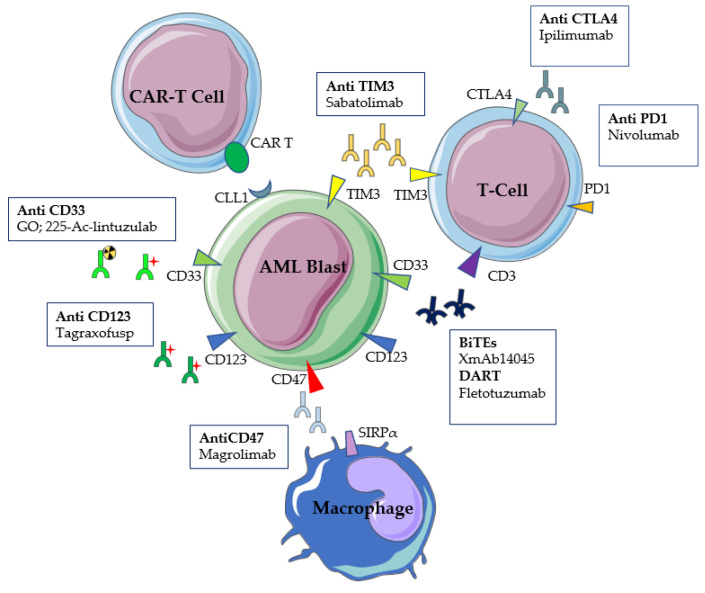
Harnessing immunity against AML blasts. Antibodies, checkpoint inhibitors, bispecific antibodies, and CAR-T cells are possible strategies in leukemia treatment.

A more detailed knowledge of the evasion mechanisms carried out by cancer cells to escape antitumor immunity, together with technical advances in the production of monoclonal antibodies (mAbs), led to the conception of a plethora of drugs specifically directed to harness immunity against AML cells (Figure 2).

These comprise gemtuzumab ozogamycin, the first antibody–drug conjugate (ADC) targeting CD33, new anti-CD33s mAbs, mAbs targeting CD123, and radiolabeled mAbs. 

Checkpoint inhibitors (anti-CTLA4 and anti-PD1-PDL1), which are already largely employed in solid oncology, have also been tested in the AML setting and may represent an interesting treatment in combination with other agents. Alternative molecules that are able to restore immune cytotoxic effect, such as sabatolimab, an anti-T cell immunoglobulin and mucin domain 3 (TIM3), and magrolimab, an anti-CD47 macrophage checkpoint inhibitor, have recently made their appearance in early phase clinical trials.

Finally, some bispecific T-cell engager (BiTE) antibodies and chimeric antigen receptor T (CAR-T) cells adapted to myeloid leukemia targets are currently being developed.

### 4.1. Monoclonal Antibodies (mAb) Anti-CD33 

CD33 (or siglec 3) is a transmembrane receptor expressed by myeloid cells that is present on the surface of AML blasts in 85–90% of adult and pediatric AML patients [82].

Gemtuzumab ozogamicin (GO) is an ADC-targeting CD33 associated with calicheamicin, a cytotoxic component that is released into target cells [83,84,85].

After an accelerated approval by the FDA for R/R AML in 2000, GO was withdrawn in 2010 due to the absence of benefit and excessive toxicity in addition to standard induction chemotherapy in the phase III study SWOG-S0106 [86,87,88,89,90].

More recently, a fractionated administration schedule allowed the relocation of GO as a therapeutic option in AML [91]. In the ALFA-0701 trial (NCT00927498), fractionated GO (3 mg/m^2^ on day 1-4-7 of induction and day 1 of consolidation treatment) in association with standard chemotherapy in adult ND (50–70 years old) AML patients resulted in an EFS gain (13.6 versus 8.8 months), despite a slightly higher early mortality (4% vs. 2%) due to hemorrhage and VOD (5%) in the GO arm compared to the control arm.

An advantage in EFS, OS, and relapse-free survival (RFS) for low and intermediate-risk cytogenetic AML groups was confirmed by a meta-analysis including 3300 patients from five randomized controlled trials that corroborated the clinical benefit of GO in these categories of patients [91].

Potential GO toxicity was considered to be outweighed by the gain in EFS, leading to the reapproval of the drug by the FDA in 2017 for newly-diagnosed CD33-positive AML in adults and relapsed or refractory CD33-positive AML in adults and pediatric patients aged 2 years and older.

The EMA approved GO for adults (>15 years old) with de novo CD33+ AML in combination with daunorubicin and cytarabine.

#### 4.1.1. New Perspectives on Gemtuzumab Ozogamycin

Several phase I and II studies are currently exploring the benefit of associating GO with other drugs, such as azacitidine, CPX-351, venetoclax, and other conventional drugs, in the induction phase of treatment.

In a retrospective study on 17 R/R AML patients, the combination of GO and azacitidine provided an overall response rate of 76.9%, emerging as a possible bridge to transplant treatment [92].

GO plus venetoclax was revealed to be safe in a phase Ib trial and the effectiveness of this combination is currently under investigation [93].

The combination of GO with the poly (ADP-ribose) polymerase inhibitors olaparib and talazoparib showed an intense antileukemic effect in preclinical models and could be included in novel combinations suitable for clinical trials in the near future [94,95,96].

In the phase I MOSAIC trial, GO was associated with midostaurin and standard 3 + 7 chemotherapy in upfront AML with a cCR of 91% [97]. 

Finally, advances in cellular therapy may open new opportunities for GO. Currently, an ongoing trial is evaluating post-transplant GO administration after an allogeneic engineered hematopoietic stem cell transplant lacking CD33 (NCT04849910).

Next generation anti-CD33s are under development, including vadastuximab talirine (SNG33A), a humanized murine anti-CD33 IgG1 mAb coupled with two molecules of pyrrolobenzodiazepine, which showed good tolerability in monotherapy and an encouraging CCR (CR + CRi 73%) in a study on 53 elderly AML patients who were ineligible for IC [98,99].

#### 4.1.2. Anti-CD123—Tagraxofusp 

CD123 is a component of the interleukin 3 receptor (IL-3Rα) expressed by hematopoietic stem cells that transmit, through the JAK2/STAT pathway, a signal for survival and proliferation [100].

CD123 has been found to be extensively overexpressed not only in AML but also in other hematological malignancies, such as blastic plasmacytoid dendritic cell neoplasm (BPDCN), MDS, systemic mastocytosis, chronic myeloid leukemia (CML), acute lymphoblastic leukemia (ALL), and hairy cell leukemia (HCL) [101,102,103,104,105,106,107].

Tagraxofusp (SL-401) is an anti-CD123 antibody conjugated to diphtheria toxin that has shown efficacy in the treatment of BPDCN [108].

In a phase I study, SL-401 was employed in 45 AML patients and showed a good safety profile but limited efficacy [109].

The combination of tagraxofusp with azacitidine or with azacitidine and venetoclax was tested in a phase Ib trial in AML, MDS, and BPDCN patients. Twelve AML patients received doublet and 14 AML patients triplet therapy, with CR or CRi in eight ND AML patients. No R/R AML patients responded in either of the two groups [110]. 

#### 4.1.3. Radiolabeled Monoclonal Antibodies

Radiolabeled mAbs can deliver ionizing radiation to target cells in a far more precise manner than the classical irradiation technique, leading to the development of different mAbs labeled with radioisotopes that decay via the emission of alpha or beta particles [111].

While humanized, the unconjugated anti-CD33 mAb lintuzumab did not confer a survival benefit in AML treatment as monotherapy and in association with IC; it has been employed for the delivery of radionuclides [112,113,114].

The radioconjugate α-emitters bismuth-213 (213Bi-) and actinium-225 (225Ac-) conjugated with lintuzumab have shown antileukemic efficacy as single agents in R/R AML and in association with cytarabine [115,116,117]. Moreover, 225Ac–lintuzumab has been shown to reverse resistance to venetoclax in AML models. A phase I/II study of venetoclax and lintuzumab–225Ac in R/R AML patients is currently ongoing (NCT03867682) [118].

### 4.2. Checkpoint Inhibitors 

Regulatory T cells expressing PD1/TIM3 are increased in leukemic bone marrow and AML blasts have been found to express PDL1, making T cell harnessing through checkpoint inhibitors (CPI) an attractive therapeutic strategy in AML [119].

#### 4.2.1. Anti-CTLA4 and Anti-PD1

The anti-CTLA4 agent ipilimumab was explored in a phase I study on 12 R/R AML patients post ASCT; it allowed a complete response lasting greater than one year in five cases, despite reported immune-mediated toxic effects and graft versus host disease (GVHD) [120]. 

While the antileukemic activity of PD1 inhibition alone seems to be low, the observation that HMAs, among their immunomodulatory effects, induce an increased expression of inhibitory molecules (PD1 and CTLA4), provided the rational to obtain a synergistic effect by combining HMAs and CPI [121,122].

The combination of nivolumab and azacitidine seems to be more effective for R/R AML patients in early salvage conditions (patients with less than two prior lines of therapy) compared to HMA alone. In this setting, in a non-randomized, open-label, phase II study, Daver and colleagues reported a median OS of 10.5 months and a 12-month OS of 50% [123]. 

Encouraging results have also been reported for the combination of azacitidine and pembrolizumab, which was tested in a phase II trial in R/R and ND AML patients greater than 65 years old, with a particularly notable efficacy in the ND setting (CCR = 47%, 8/22 pts) [124].

The triplet consisting of azacitidine, nivolumab, and ipilimumab was shown to slightly improve OS in R/R AML patients. Single-cell immunophenotype profiling could help in selecting and predicting the response to CPI [125,126].

#### 4.2.2. Anti-T Cell Immunoglobulin and Mucin Domain 3 (TIM3)

T-cell immunoglobulin and mucin domain 3 (TIM3) is a transmembrane co-inhibitor receptor expressed by T cells that leads to T-cell exhaustion [127]. 

The blockage of TIM3 and PD1 with antibodies improves the anti-cancer T cell response in vitro and in murine models [128,129].

Galectin-9, a TIM3 ligand, is produced by leukemia stem cells (LSCs) and protects them from T cell targeting. Moreover, LSCs—but not hematopoietic stem cells (HSCs)—have been found to express TIM3, which seems to be activated in an autocrine manner, leading to the accumulation of beta catenin in the intracellular compartment and the promotion of a self-renewal signal [130].

In fact, sabatolimab, a humanized IgG4 anti TIM3 mAb, is the only TIM3 inhibitor currently being studied in AML and MDS clinical trials in monotherapy or in combination with other drugs (e.g., venetoclax, HMAs); it has shown promising response rates and manageable toxicities [131].

### 4.3. Anti-CD47 Antibodies—Magrolimab

CD47 is a transmembrane protein that transmits a “do not eat me” signal. CD47 expression counterbalances pro-phagocytosis signals in malignant cells, allowing for the evasion of macrophage phagocytosis [132]. 

**Figure 3:**
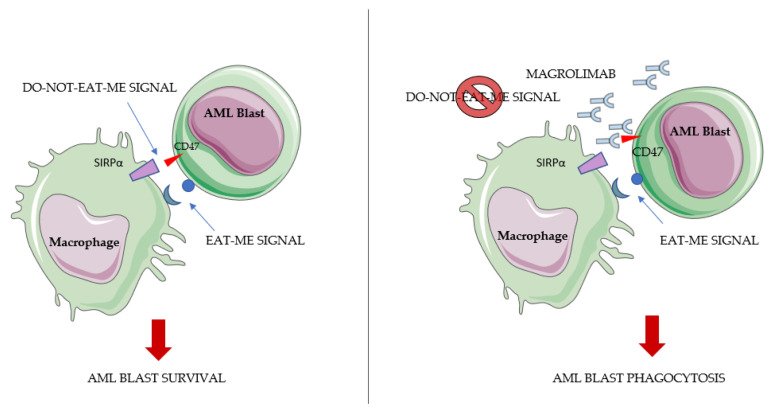
AML blasts avoid phagocytosis through CD47–SIRPα interaction, transmitting a ‘do-not-eat-me’ signal that cancels the malignant cell’s ‘eat-me’ signal expression. Magrolimab (anti-CD47) interferes with the CD47 and SIRPα interaction, abolishing this escape mechanism and inducing blast phagocytosis.

CD47 is expressed in AML cells and its inhibition has shown a capacity for eliminating LSCs in preclinical models (Figure 3) [133].

The IgG4 anti-CD47 magrolimab showed a satisfactory tolerability profile with limited antileukemic activity as monotherapy in R/R AML patients in a phase I trial [134]. 

The observation that azacitidine upregulates the ‘eat me’ signal on leukemic cells, increasing magrolimab efficacy, provided the rationale for testing their association [135].

In a recent phase Ib trial, the combination of magrolimab with azacitidine showed a 64% and 91% CCR in untreated AML and high risk MDS patients non eligible for IC, respectively. Interestingly, 74% of CCR was found in the TP53-mutated AML subgroup [136].

A phase III study evaluating the combination of magrolimab with azacitidine (NCT04778397) and a phase I study evaluating the combination magrolimab, azacytidine, and venetoclax are currently ongoing. (NCT04435691).

### 4.4. Bi-Specific T-Cell Engagers (BiTEs)

Bispecific antibodies are compounds capable of binding two different targets, usually expressed by different cells. Particularly, bispecific T-cell engagers (BiTEs) are agents whose action is triggered by the engagement of both antigen sites, determining T-cell activation with subsequent killing of the target cell and increased pro-inflammatory cytokine release, independent of the costimulatory signal [137,138].

This technology started to be investigated more than 50 years ago; however, its clinical development and application in a hematological setting is much more recent and is mainly related to lymphoproliferative disorders [139,140,141].

The first bispecific antibody approved for use in a hematological malignancy was blinatumomab, which granted the indication for treatment of relapsed or refractory (R/R) acute lymphoblastic leukemia (ALL) in 2014.

The first BiTE designed and employed for AML treatment was AMG330, which targets the antigens CD33 on tumor cells and CD3 on T cells; preliminary data showed important in vitro antileukemic activity and particularly the capacity of avoiding key mechanisms for tumor resistance, such as CD33 downregulation [142]. 

The preliminary results of a phase I trial with AMG330 for R/R AML showed an ORR of 19% with an acceptable toxicity profile characterized mostly by cytokine release syndrome (CRS; 67% all grades, 13% grade 3–4) and an AE correlating with blast count at the baseline and dose level. This drug presents a short half-life of approximately two hours, needing continuous intravenous administration [143].

A modified CD33/CD3 BiTE is AMG673, which presents a longer half-life than AMG330, permitting an intermittent schedule of intravenous administration. Efficacy data reported in the phase I trial showed a decrease in blast count in 44% of evaluable patients; however, 50% of patients experienced CRS (27% of grade 3, no grade 4 observed) [144]. Other CD33/CD3 BiTEs currently under investigation are AMV564, which seems to be correlated with less CRS in the preliminary data of a phase I trial, and JNJ-67371244, for which no data are available yet [145].

Vibecotamab (XmAb14045) has been conceived to target CD3 and CD123 and was tested in a phase I study in 63 heavily pre-treated R/R AML patients.

Efficacy was reported only for the two highest dose level studies in the trial (1.3 and 2.3 µg/kg weekly) with CR/CRi in three of thirteen AML patients, of which two could proceed to transplant.

The main toxicities were CRS (77%, with 11% of grade ≥ 3), fatigue, neutropenic fever, and peripheral edema [146].

A different type of molecule called DART (dual-affinity re-targeting) was recently developed; it differs from BiTE due to the presence of variable domains of heavy and light chains which engage the target antigens on two separate polypeptides [147]. This configuration provides a more stable molecule due to the presence of an additional disulfide bridge and seems to also give an advantage in terms of efficacy when compared to the BiTE format [148].

The first DART investigated in AML was flotetuzumab (MGD006 or S80880), which binds to CD3 and CD123; the phase I/II trial showed a CR/CRi rate of 27% and a median OS of 10.2 months, independent from cytogenetic risk category. Grade 3 CRS was observed in 8% of patients and mostly reversible and frequently treated with the early employment of tocilizumab. The recommended dose was 500 ng/kg/day by continuous intravenous administration [149].

### 4.5. Chimeric Antigen Receptor T Cells (CAR-Ts)

One of the most innovative approaches in modern oncohematologic immunotherapy is represented by chimeric antigen receptor T cells (CAR-Ts). This technology is based on engineered synthetic receptors permitting the regulation of T cell activity to recognize and eliminate neoplastic cells expressing a specific target antigen. The induced activity of CARs is independent from the presence of co-stimulatory proteins or MHC receptors [150]. 

Despite its great impact in lymphoproliferative disorders, leading to the approval of several CAR-T compounds by EMA and FDA in the last years, the development of this immunotherapy for AML treatment has been slower and less successful, due to adverse microenvironmental conditions and the reduced number of target antigens selectively expressed by AML cells, determining suboptimal efficacy and increased toxicity [151].

Two promising target antigens are CD33, which is highly expressed on both AML and healthy stem cells, and CLL-1 (or CLEC12A), a more selective agent for leukemic blasts. In the first phase I trial using dual CD33-CLL-1 CAR-T cells on 9 R/R AML patients, a 4-week evaluation reported an interesting MRD negative rate of 78%, using flow cytometry, with six patients proceeding to ASCT. CRS was common, but was mostly of grade 1–2 (75% of cases) and efficaciously managed with corticosteroid treatment [152]. 

Another widely studied AML antigen is CD123; in a phase I trial using a CD123 CAR-T cell treatment, the data showed significant antileukemic activity with manageable CRS [153]. CD38 CAR-T cells are currently under investigation in a phase I/II trial: this antigen, well known for its implication in plasma cell dyscrasias, has also been found to be highly expressed in AML blasts [154].

In a phase I/II trial with six patients, four of them achieved CR or CRi, with a median PFS of 6.4 months. CRS was highly manageable, mostly of grade 1–2 (83% of patients) [155].

A further improvement of CAR-T technology is the universal CAR-T platform (UniCAR): this CAR is designed to recognize a peptide motif included in the second component of the molecule, called the targeting module, which confers specificity against the antigen of choice. Three AML patients were treated with UniCAR-T-CD123 with encouraging efficacy (one PR, two CRi) and toxicity (two cases of grade 1 CRS) results [156].

## 5. New Formulations, Old Drugs

### 5.1. CPX-351 (Vyxeos)

CPX-351 is a liposomal formulation with a 1:5 molecular ratio of daunorubicin and cytarabine that optimizes drug delivery, with preferential uptake by LSCs compared to normal HSCs [157,158,159].

In a phase III trial including 309 patients from 60 to 75 years old with ND high-risk AML, CPX-351 showed a significantly improved median OS and higher ORR compared to the classic 7 + 3 approach, despite a longer time to neutrophil and platelet count recovery [160]. CPX-351 was approved by the FDA and EMA for ND and therapy-related (TR) AML, and real-world data confirmed the efficacy of CPX-351 in monotherapy, with promising outcomes after HSCT [161].

Ongoing trials are exploring the role of CPX-351 beyond TR-AML (MDS, LMMC, myelofibrosis, and myeloproliferative neoplasms) and its associations with other drugs (venetoclax, gilteritinib, palbociclib, glasdegib, and GO).

### 5.2. Oral Azacitidine (Oral-Aza)

The well-known HMA azacitidine has been recently developed in an oral form, which is administered in an extended doses schedule of 14 or 21 days, that exercises anti tumoral activity through hypomethylating and immunomodulatory effects which are not yet fully understood [162,163].

The oral formulation of azacitidine allows a prolonged schedule of administration, which consequently extends the exposure of AML cells to the drug, sparing inconveniences related to injections and promoting a particular hypomethylating profile and weaker cytotoxic effects [164,165,166,167].

A phase I study showed that oral-aza, administered daily for 14- and 21-day affords 38% and 57% greater azacitidine exposure, respectively, in comparison with the classical injectable schedule (75 mg/m^2^ for 7 consecutive days) [168,169].

An ORR of 22% in AML patients was reported by phase II AZA-MDS-004 study, in which oral-aza was administered for 21 days [170].

Nowadays, the approval of oral azacitidine in the EU, USA, and Canada for AML patients in RC1 or RCi1 after IC who are not eligible for ASCT is based on the results provided by the QUAZAR AML-001 study. In this trial, oral-aza maintenance after IC significantly prolonged OS (24.7 versus 14.8 months) and RFS (10.2 versus 4.8 months) in AML patients older than 55 years, compared to a placebo [171].

## 6. Tyrosine Kinase Inhibitors (TKIs) and the RAS Pathway Inhibitors

### 6.1. FLT3 Inhibitors

**Figure 4:**
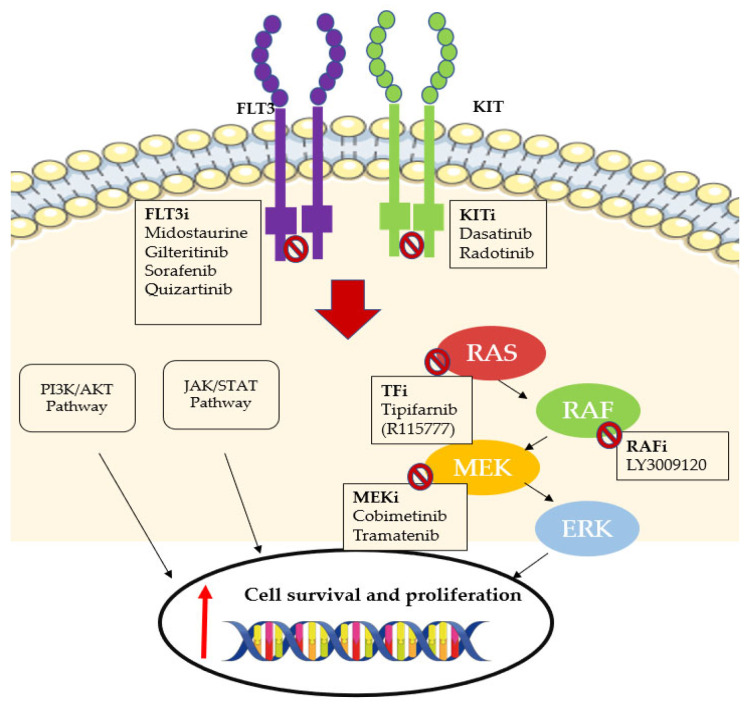
FLT3 and KIT inhibitors and the RAS-RAF-MEK-ERK signaling pathway and its inhibitors.

Over the last two decades, several TKIs have been developed to treat patients with FLT3 gene mutations (Figure 4). Approximately 30% of AML patients carry a mutation at diagnosis, whether an internal tandem duplication (FLT3-ITD) or a point mutation in the tyrosine kinase domain (FLT3-TKD). These patients are historically associated with a bad prognosis, a high risk of relapse, and low cure rates. FLT3is have been evaluated in frontline treatment, salvage settings, or as maintenance treatment after ASCT [172].

New clinical trials are now exploring the use of first and second-generation FLT3is in association with non-intensive treatments in frontline or R/R settings and the use of second-generation FLT3is in association with IC in a frontline setting. 

#### 6.1.1. Midostaurin

Midostaurin is a first-generation FLT3i now approved by the FDA and EMA in association with standard IC for ND AML based on the results from the RATIFY Trial [173]. 

The trial randomized 717 patients to receive classic IC and midostaurin or classic IC and a placebo. The CR rate was 58.9% and 53.6% while the median 4-year OS was 51.4% and 44.3% in the midostaurin and placebo groups, respectively. The safety profile was similar in both groups, and this has recently been confirmed in the RADIUS-X expanded access program [174].

Midostaurin in association with azacitidine was evaluated in 54 patients in a phase I/II study of AML and MDS unfit for IC or R/R after previous treatments: the ORR was 26%, with a CR + CRi rate of 13%. The median OS was 22 weeks [175].

#### 6.1.2. Sorafenib

Sorafenib is another first-generation FLT3i. It has been tested in phase II and III trials in ND AML in association with IC with discordant outcomes [176,177].

Very interesting results have been published in the post-transplant setting. The SORMAIN study is the only prospective RCT in this setting, randomizing 83 adult patients with FLT3-ITD AML in CR post ASCT to receive sorafenib or a placebo. The 2-year RFS was 85% and 53.3% in the sorafenib and placebo groups, respectively (*p* = 0.013), and the OS was significantly longer in the sorafenib arm (*p* = 0.03) [178]. 

#### 6.1.3. Gilteritinib

Gilterinib is a highly selective second-generation FLT3i with activity against AML cells harboring FLT3-ITD and TKD mutations. Significant results in R/R patients lead to its experimental use in the frontline setting.

The phase III ADMIRAL trial led to the approval of gilteritinib in monotherapy in R/R FLT3-mutated patients. Gilteritinib was compared to salvage chemotherapy in 371 patients. The median OS was improved in the gilteritinib arm (9.3 vs. 5.6 months; *p* < 0.001), as was the CR rate (34% vs. 15.3%). It should be noted that 88% of patients were not pre-exposed to other FLT3is [179].

The phase III trial LACEWING study (NCT02752035) evaluated gilteritinib with azacitidine compared to azacitidine alone in ND FLT3-mutated AML patients who were ineligible for IC. Higher CRc rates were observed in the experimental arm, but a similar OS was reported in both arms [180].

As already discussed, gilterinib has been tested in association with venetoclax and in triplet with azacitidine and venetoclax for R/R and ND AML patients with interesting results [42,43]. 

#### 6.1.4. Quizartinib 

In the QUANTUM-R phase III study, which compared the second-generation FLT3 inhibitor quizartinib to salvage chemotherapy in R/R FLT3-ITD AML, quizartinib showed a survival benefit with an OS of 6.2 months compared to 4.7 in the control arm [181].

Quizartinib is currently approved in Japan for the treatment of R/R FLT3-ITD-mutated AML. 

In a phase I/II trial, the combinations of quizartinib with azacitidine or LDAC were evaluated in ND or RR FLT3-ITD MDS-AML patients, producing a median OS of 19.2 months (quizartinib/azacitidine) and 8.5 months (quizartinib/LDAC) in the frontline setting and 10.5 months (quizartinib/azacitidine) and 6.4 months (quizartinib/LDAC) in the R/R setting [182].

Quizartinib was also evaluated in a triplet therapy in association with azacitidine and venetoclax in ND and R/R AML FLT3-ITD-mutated patients who were ineligible for IC. All five patients in the ND cohort achieved CRc. The CRc rate among R/R patients was 65%, with an encouraging OS of 7.5 months and a 1-year OS of 34%. It should be noted that 68% of these patients received gilteritinib and that RAS/MAPK mutations were associated with primary and secondary resistance [183].

### 6.2. KIT Inhibitors

The KIT gene encodes for a membrane receptor tyrosine kinase (CD117) that is frequently mutated in core-binding factor AML, suggesting a possible role of KIT inhibition in AML treatment (Figure 4) [184,185].

The BCR/ABL inhibitors dasatinib and radotinib have been shown to promote AML cell death by targeting c-KIT in AML cell lines and murine models, suggesting a potential role in clinical application [186,187].

### 6.3. RAS Pathway Inhibitors

Rat sarcoma (RAS) proto-oncogenic proteins have been largely studied in recent decades, and RAS mutations have been found to play a role in about 30% of human cancers [188].

The three highly homologous RAS proteins (KRAS, NRAS, and HRAS) are activated through a GTP phosphorylation process and promote cell survival and proliferation through the RAS/RAF/MEK/ERK pathway (Figure 4) [189].

RAS mutations are found in approximately 15–40% of AML diagnoses, and the most frequent mutated RAS protein in myeloid malignancies is NRAS [190,191,192,193,194,195].

Once synthesized, RAS proteins undergo cytosolic modifications before being collocated to the membrane surface where they are effective. One of these modifications is prenylation, which requires an enzyme called farnesyltransferase (FT) [196].

Tipifarnib (R115777) is an inhibitor of FT that has been tested in R/R AML, with reported clinical responses in 29% of patients (10 out on 34) with two CR [197,198].

In phase I studies, tipifarnib was shown to be safe in combination with IC and with bortezomib in patients with ND AML [199].

To target the RAS pathway, a RAF inhibitor has also been developed (LY3009120) and has shown synergistic proprieties with cytarabine in RAS-mutated AML cell lines [200].

Acting on the same signal transmission pathway, some MEK inhibitors have been developed and studied in early phase trials. Their clinical development is still at an immature stage and their role will likely be in combination with other agents. 

The MEK inhibitor binimetinib has been shown to be safe in RAS-mutated AML, with very limited activity as monotherapy and in combination with the ATP-competitive pan-AKT inhibitor GSK2141795 (targeting the PI3K/PTEN/AKT/mTOR pathway) [201,202].

Trametinib is another MEK inhibitor that showed activity in RAS-mutated AML in a phase I/II study [203].

Cobimetinib, a third MEK inhibitor, is currently being studied in combination with venetoclax [204].

Downstream in the same pathway, ERK activation was shown to confer resistance to TKI; the association of trametinib with midostaurin showed a synergistic effect and could emerge as a new exploitable strategy in FLT3-mutated AML patients [205,206].

## 7. Isocitrate Dehydrogenase Inhibitors

IDHs belong to a group of enzymes that catalyze the oxidative decarboxylation of isocitrate to alfa-ketoglutarate (α-KG), generating NADPH. 

Mutations involving arginine in the active site of IDH1/2 (R132 in IDH1 and R140 or R172 in IDH2) result in mutant IDH1/2 proteins that have acquired the capability of converting α-ketoglutarate in 2-hydroxyglutarate (2-HG) [207,208].

**Figure 5:**
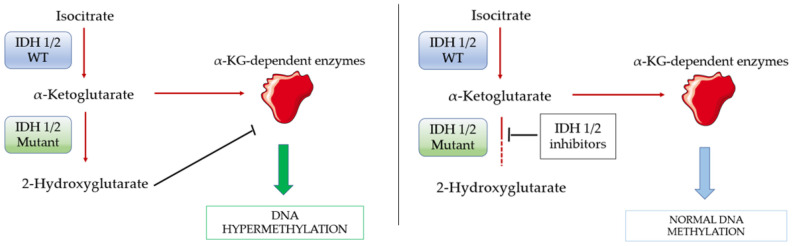
IDH1/2 mutant converts α-ketoglutarate into 2-hydroxyglutarate, which interacts with α-KG-dependent enzymes and leads to DNA hypermethylation. IDH1/2 inhibitors prevent α-KG production and restore a normal DNA methylation profile.

2-HG acts as a competitive inhibitor of α-KG, interfering with α-KG-dependent enzymes, which play a role in several metabolic processes and DNA methylation, and produces a characteristic hypermethylated DNA phenotype (Figure 5) [209,210].

IDH1 and IDH2 mutations are found in approximately 8% and 12% of AML cases, respectively, frequently co-occurring with FLT3/NPM1 mutations [211,212].

It should be noted that IDH mutations have been also described in other malignancies (glioblastoma, chondrosarcoma, cholangiocarcinoma, and angioimmunoblastic T cell lymphoma) [209,213,214,215].

The prognostic impact of IDH1/2 mutations in AML is still a matter of debate [216].

Enasidenib (AG-221) and ivosidenib (AG-120), which are IDH2 and IDH1 inhibitors, respectively, target IDH-mutated proteins.

Enasidenib was proven to be well tolerated and safe as a single agent and capable of inducing responses in R/R IDH2-mutated AML patients in a phase I/II study, with an ORR of 40.3% and a median DOR of 5.8 months [217]. These results led to enasidenib approval for R/R IDH2-mutated AML in the USA. 

Unfortunately, the phase III trial IDHENTIFY (ClinicalTrials.gov (accessed on 7 February 2022), NCT02577406), which compared enasidenib to conventional treatments (best supportive care alone or in combination with azacytidine or low-dose or intermediate dose cytarabine) in R/R IDH2-mutated AML patients was announced to have missed the primary endpoint of OS in the enasidenib arm [218].

In an ND setting, a phase I/II clinical trial investigating enasidenib monotherapy in 39 ND IDH2-mutated AML patients reported an ORR of 30.8% with a CCR of 21% and a median OS of 11.3 months [219].

Ivosidenib monotherapy was tested in a phase I study in 268 IDH1-mutated AML patients, with 179 patients in the R/R setting. CR + CRi was obtained in 30.4% of treated patients, with a global DOR of 6.5 months that reached 9.3 months in CR patients and a low rate of AEs of grade 3–4 (mainly QT prolongation in 7.8%, IDH differentiation syndrome in 3.9%, and thrombocytopenia in 3.4% of cases) [220].

Resistance to ivosidenib has been found to be correlated with receptor tyrosine kinase pathway mutations in this population of R/R AML patients [221]. 

Both in the ND and R/R settings, enasidenib and ivosidenib are under investigation in association with other agents.

Compared with azacitidine alone, Dinardo et al. showed that the combination of enasidenib with azacitidine resulted in a better and greater-than-additive effect on ORR (74% versus 36%) and a better CRR (54% versus 12%) [222].

The safety and clinical activity of the combination of ivosidenib with azacitidine was evaluated in a phase b study in patients with ND IDH1-mutated AML who were ineligible for IC.

This association, beside good tolerance without dose-limiting toxicities, reported interesting and encouraging results concerning clinical efficacy, with an ORR of 78.3%, a CR rate of 61%, and a median time to first response of 1.8 months (range, 0.7–3.8 months); the median duration of CR, CR/CRh, and overall response were not reached [223].

The association of both agents with standard IC in ND AML in a phase I study did not delay hematological recovery compared with historical data. Ivosidenib-treated and enasidenib-treated patients had encouraging 12-month survival probabilities of >75%, considering the high portion of secondary AML (~30%) and a majority of elderly patients (more than 60 years old) in the study population. Phase III studies are currently ongoing to further evaluate the association of IDH inhibitors with IC [224].

In the phase Ib/II enaven-AML trial, enasidenib was associated with venetoclax in 11 RR/AML very high-risk (VHR) MDS patients with an encouraging ORR (55%) [49].

Multiple combinations of enasidenib/ivosidenib with venetoclax (NCT04092179), decitabine (NCT05010772), decitabine and venetoclax (NCT04774393), and classical induction/consolidation therapy (NCT03839771) are currently under investigation along with the role of IDH inhibitors in maintenance therapy following ASCT in AML (NCT03728335, NCT04522895); in IDH2-mutated MDS (NCT03744390); in association with CPX-351, fedratinib (NCT04955938), and ruxolitinib (NCT04281498); and in IDH1/2-mutated high risk myeloproliferative syndromes. 

## 8. Others

### 8.1. Hedgehog Inhibitors—Glasdegib

The hedgehog signaling pathway plays an essential role in embryonal and adult hematopoiesis and its aberrant activation has been found to drive hematological malignancies, including AML [225,226,227].

In the phase II BRIGHT AML 1003 trial, the combination of glasdegib and LDAC improved CR rate (17 versus 2.3%) and median OS (8.8 versus 4.9 months) compared to LDAC alone in a population of AML and high-risk MDS patients [228].

These results led to the FDA and EMA approval of glasdegib in combination with LDAC in AML patients aged more than 75 years who are ineligible for IC [229].

Moreover, the long-term BRIGHT AML 1003 analysis showed a survival advantage of the association of glasdegib with LDAC versus LDAC alone, with a more pronounced benefit in secondary AML [230].

Ongoing trials are currently investigating new associations of glasdegib with azacitidine and IC with cytarabine/daunorubicin (NCT03416179, NCT02367456, and NCT01546038).

### 8.2. IRAK4 Inhibitors

IRAK4 (interleukin-1 receptor-associated kinase 4) in association with IRAK1 can activate NF-κB pathway signaling, triggering cell survival. IRAK4-L RNA isoform expression, generated by alternating splicing promoted by mutant U2AF1 splicing factor, has been found to be associated with AML and is required for leukemic cell function [231].

The inhibition of IRAK-4 through the IRAK-kinase inhibitor CA-4948 was shown to arrest leukemic growth in AML cells expressing IRAK4-L and to limit the spread of THP1 leukemic cells in a xenograft murine model [231].

Currently, the oral IRAK-4 inhibitor CA-4948 is under investigation in monotherapy and in combination with azacitidine or venetoclax in R/R AML and R/R MDS settings (NCT04278768).

### 8.3. Menin-KMT2A (MLL) Inhibitor

The histone-lysine-N-methyltransferase 2A (KMT2A) gene and menin-1 gene are part of a complex that plays an essential role in regulating the expression of homeobox genes. Mutations in KMT2A are responsible for aberrant Hox gene expression leading to leukemogenesis [232,233].

Accounting for the 5–10% of AML diagnoses, DNMT3A-mutated cases constitute a very poor prognostic group of disease [234].

The menin inhibitor MI-3454 was revealed to block proliferation and induce differentiation in AML cells and remission in AML KMT3A/NPM1 mutated murine models [235].

Indeed, the menin inhibitor KO-539 is under investigation in the KOMET-001 phase I/II study in R/R AML patients [236].

## 9. Discussion and Conclusions

Until recent years, AML has been treated with IC followed by ASCT for fit patients with the intention to cure, and with HMA for patients ineligible for IC, mainly with an intent to improve quality of life. A significant portion of more fragile patients could also be oriented towards pure palliative care.

The absence of valid alternative treatments outside clinical trials leaves little room for therapeutical strategy in frontline treatment and, in the R/R setting, if remission cannot be obtained with salvage chemotherapy to proceed to transplant, supportive care should be encouraged. 

Recent advances in the understanding of the molecular basis of leukemogenesis and the mechanisms sustaining LSC survival has allowed a better characterization of AML disease and a more precise prognostic stratification. 

With the therapeutical plan, these advances led to the development of several classes of agents with different mechanisms of action, opening opportunities for new hopes for patients and prompting new management possibilities for a disease treated for many years in a single way.

Nowadays, IDH1-2 inhibitors, FLT3is, and GO are frequently incorporated in AML treatment according to a tailored approach; future challenges include assessing their role in new combinations and the development of new, more potent, and better tolerated next-generation molecules. New formulations of drugs (CPX-351 and oral azacitidine) with particularly interesting pharmacokinetic and tolerability profiles are also currently available and widely used.

Among recently approved drugs, the BCL-2 inhibitor venetoclax is one of the most interesting agents, even if its role in treating AML is still largely unexplored. We know that in combination with HMAs and other agents, is a valuable upfront treatment component for unfit and fit patients; however, new combinations carrying promising results in early phase trials are currently under investigation. 

Beyond induction, venetoclax may keep an interesting role in consolidation and maintenance; however, again, appropriate schedules and more advantageous combinations need to be established.

Following the path that has been traced by venetoclax, new classes of drugs will likely enrich the therapeutical landscape for AML, from which every population of patients could benefit, opening the pathway for several different treatment strategies. 

In this race, agents harnessing the intrinsic apoptotic pathway and their associations, target therapies, and mAbs are progressing more rapidly; however, owing to the acquired knowledge in lymphoproliferative disease, BiTEs and CAR-T cells could also gain ground in the next years and may turn out to be key weapons against AML. 

Frail patients could particularly benefit from the synergic association of new molecules without excessive side effects, owing to different and non-overlapping toxicity profiles of new single agents, allowing for globally better outcomes in terms of treatment efficacy and quality of life. Moreover, a larger portion of patients with preserved performance status could proceed to transplant, with expected advantages in long-term survival, questioning the optimal employment of new drugs in the maintenance post-transplant setting. 

On the other hand, the incorporation of new agents in IC schedules represents a hope for deeper and long-lasting responses in fit patients, maybe questioning the role of transplant for some AML patients in the future.

Moreover, several articulated sequential strategies combining different agents at different moments (induction, consolidation, and maintenance) of treatment could be imagined. Defining which is the best place for every given agent is another challenge to deal with.

No less important, on the bases of molecular and cytogenetic disease characteristics together with a better understanding in molecular biology and the immunology of LSCs, specific disease-tailored treatment could emerge.

Complex karyotype disease and very bad prognosis mutations (such as EVI1 and TP53) characterize a population that is still difficult to treat with very poor outcomes, constituting an unmet therapeutical need. More effective drugs for such poor-prognosis disease should be determined and new agents will perhaps represent interesting therapeutic options.

Undoubtedly, there is an unmet necessity for curative therapy in AML; access to new drugs should be encouraged, and well-designed, large-scale trials are needed to assess the efficacy of new molecules and to trace patient-tailored strategy. Observational data will be essential to support and to confirm clinical trial results in the real world AML population and, moreover, to provide survival outcomes on a longer-term follow-up.

In conclusion, important and exciting progress in recent years is offering new therapeutical possibilities, projecting AML treatment into a precision medicine dimension, which is disease and patient tailored and capable of improving life quality and life expectancy for most patients. 

## Figures and Tables

**Table 1:** List of studies assessing Venetoclax in association with other agents in acute myeloid leukemia. AZA—azacitidine; BETi—BET inhibitor; CLA—cladribine; CCML—chronic myelomonocytic leukemia; COB—cobimetinib; DEC—decitabine; ENA—enasidenib; FLAG—fludarabine; G-CSF—Granulocyte colony-stimulating factor; GILT—gilteritinib; HIDAC—high-dose cytarabine; HMA—hypomethylating agent; IDA—idarubicin; IDASA—idasanutlin; IVO—ivosidenib; LINT-AC225—lintuzumab-Ac225; LDAC—low dose cytarabine; MAGRO—magrolimab; MCL1i—MCL1 inhibitor; MDS—myelodysplastic syndrome; MEKi—MEK inhibitor; MIDO—midostaurin; MIVE—mivebresib; SAB—sabatolimab; SORA—sorafenib; TAGR—tagraxofusp; VEN—venetoclax.

Agents in Combination with VEN	Population	Phase Study	References
***VEN plus HMA/LDAC***			
*AZA*	ND AML ineligible for IC	3	27
*LDAC*	ND AML ineligible for IC	3	33
***Intensive Chemotherapy***			
*7 + 3*	ND AML	1b	NCT03709758
*7 + 3*	ND AML and MDS-EB	3	NCT04628026
*CLA, HIDAC, IDA*	ND AML, HR-MDS, and MPAL	2	34
*5 + 2*	ND AML ≥ 65 years old	1b	35
*FLAG-IDA*	ND and R/R-AML	1b/2	36–37
*CPX-351*	ND AML	1b	NCT04075747
***‘Non-Intensive’ Combinations***			
*GILT*	R/R FLT3 mutated AML	1	42
*AZA plus GILT*	R/R and ND AML/high risk CMML/MDS FLT3-ITD or -TKD mutated	½	43
*DEC and FLT3i (GILT/SORA/MIDO)*	ND or R/R FLT3 mutated AML	2	44
*IVO and AZA*	MDS, ND, and R/R AML IDH1+	1b/2	49
*ENA*	R/R AML IDH2+	1b/2	50
*AZA and MAGRO*	R/R AML, ND AML IC ineligible	1/2	NCT04435691
*MIVE (pan-BETi)*	R/R AML	1	NCT02391480
*S64315 (MCL1i)*	R/R Hematological malignancies	1	NCT03672695
*IDASA*	R/R AML ≥ 60 years old	1b	77
*GO*	R/R AML	1b	94
*TAGR plus AZA*	ND and R/R AML, MDS, or BPDCN	1b	111
*LINT-AC225*	R/R AML	1/2	119
*SAB*	High or very high risk MDS	2	NCT04812548
*COB (MEKi)*	R/R AML ≥ 60 years old, IC ineligible	1b	207
*CA-4948*	R/R AML and high risk MDS	1/2a	NCT04278768

## Data Availability

Not applicable.
